# Spider Movement, UV Reflectance and Size, but Not Spider Crypsis, Affect the Response of Honeybees to Australian Crab Spiders

**DOI:** 10.1371/journal.pone.0017136

**Published:** 2011-02-16

**Authors:** Ana L. Llandres, Miguel A. Rodríguez-Gironés

**Affiliations:** Department of Functional and Evolutionary Ecology, Estación Experimental de Zonas Áridas (CSIC), Almeria, Spain; University of Western Ontario, Canada

## Abstract

According to the crypsis hypothesis, the ability of female crab spiders to change body colour and match the colour of flowers has been selected because flower visitors are less likely to detect spiders that match the colour of the flowers used as hunting platform. However, recent findings suggest that spider crypsis plays a minor role in predator detection and some studies even showed that pollinators can become attracted to flowers harbouring Australian crab spider when the UV contrast between spider and flower increases. Here we studied the response of *Apis mellifera* honeybees to the presence of white or yellow *Thomisus spectabilis* Australian crab spiders sitting on *Bidens alba* inflorescences and also the response of honeybees to crab spiders that we made easily detectable painting blue their forelimbs or abdomen. To account for the visual systems of crab spider's prey, we measured the reflectance properties of the spiders and inflorescences used for the experiments. We found that honeybees did not respond to the degree of matching between spiders and inflorescences (either chromatic or achromatic contrast): they responded similarly to white and yellow spiders, to control and painted spiders. However spider UV reflection, spider size and spider movement determined honeybee behaviour: the probability that honeybees landed on spider-harbouring inflorescences was greatest when the spiders were large and had high UV reflectance or when spiders were small and reflected little UV, and honeybees were more likely to reject inflorescences if spiders moved as the bee approached the inflorescence. Our study suggests that only the large, but not the small Australian crab spiders deceive their preys by reflecting UV light, and highlights the importance of other cues that elicited an anti-predator response in honeybees.

## Introduction

Predators have evolved a wide variety of strategies to capture their prey. Among these strategies, the sit and wait tactic consists on remaining stationary and attacking approaching prey [1], [2] and it is commonly found in insects, arachnids, amphibians, lizards and snakes, among other animal groups [3]–[7]. Despite the fact that animals that present this strategy do not actively search for their food, they have evolved several tactics that can increase their chances of capturing incoming prey. To cite some examples, sit and wait predators are often under selective pressure to select profitable hunting sites [8]–[10], to present cryptic coloration to avoid being detected by their prey [11] or to attract their prey by luring them [12], [13].

Many crab spiders (Thomisidae) specialise in ambushing pollinators on flowers. In several species, adult females can change their body colour to match the colour of the flowers on which they sit [10], [14]–[16]. Moreover, some studies report that crab spiders settled preferentially on flowers that matched their body colour: yellow crab spiders selected preferentially yellow flowers and white crab spiders tended to sit on white flowers to forage [17], [18]. All these studies support the crypsis hypothesis in crab spiders, according to which the ability to change body colour to match the colour of flowers has been selected in crab spiders because flower visitors are less likely to detect spiders when they match the colour of the flower used as hunting platform [10], [14], [15].

Some studies show indeed that pollinators use visual cues to assess the presence of predators on flowers while foraging [19]–[21]. Different bee species, like *Apis mellifera* and *Trigona* sp., avoided *Rubus rosifolius* flowers containing artificial crab spiders [19]. When flowers contained objects resembling different morphological traits of spiders (abdomen or forelimbs), bees avoided objects resembling spider forelimbs [19]. Likewise, solitary bees and hover flies avoided *Anthemis tinctoria* flowers containing a pinned dried *Xysticus* sp. crab spider [20]. Different species of pollinators, however, reacted differently towards spider harbouring flowers. While some species avoided flowers with spiders, others showed indifference towards them [20]. Furthermore, at least in some systems spider colour matching with the background plays at best a minor role in predator detection [22].

Even more surprising is the finding that some pollinators can become attracted to spider-harbouring flowers when the colour contrast between spider and flower increases [23]. Australian crab spiders reflect more UV-light than their flowers, and are therefore conspicuous to bees [23]. Nevertheless, in the green house bees were attracted to UV-reflecting spiders, suggesting that Australian spiders lure prey with colours that pollinators associate with food rewards [23], [24]. European bees, *Apis mellifera,* approached and landed more on inflorescences with UV-reflecting crab spiders than on vacant inflorescences [23], [24]. This preference disappeared when UV reflection was prevented applying a UV-absorber to crab spiders, indicating that UV reflection mediates bee preference [25]. Australian native bees, *Austroplebeia australis* and *Trigona carbonaria* were also more likely to approach inflorescences harbouring UV-reflecting *Thomisus spectabilis* than vacant inflorescences, but they landed preferentially on vacant inflorescences [21], [26]. These studies suggest that in the co-evolution between Australian native bees and crab spiders, the bees have evolved an anti-predatory response. In contrast, the European honeybees, introduced into Australia in 1822 [27], have not had the opportunity to evolve a response to the deceptive Australian crab spider.

The aim of this study was to determine, under field conditions, the effect of colour matching on the interaction between the Australian crab spider *Thomisus spectabilis* and the European honeybee *Apis mellifera*. We studied the response of honeybees to the presence of white or yellow crab spiders sitting on *Bidens alba* inflorescences (white daisies with yellow centres) and also the response of honeybees to crab spiders that we made easily detectable by painting blue the spider's forelimbs or abdomen. Honeybees responded similarly to white and yellow spiders, to control and painted spiders, regardless of the morphological trait of the spider painted blue. However spider UV reflection, spider size and spider movement affected honeybee behaviour: honeybees were more likely to land on spider-harbouring inflorescences when the spiders were large and had high UV reflectance or when spiders were small and reflected little UV, than when spiders had other trait combinations. In addition, honeybees were more likely to reject inflorescences if spiders moved as the bee approached the inflorescence. Finally, spider hunting success was affected by spider size, but not by the colour attributes of the spider.

## Materials and Methods

### Ethics statement

Animal ethics permits for invertebrates are not required in Australia, nevertheless our field work protocol adheres to the ASAB ethics guidelines (http://asab.nottingham.ac.uk/ethics/guidelines.php), whereby we minimized the impact on individuals and populations by using the least disruptive technique. As all field work was completed in non-protected areas, no specific collection permits were required.

### Study area and species

**Figure 1:**
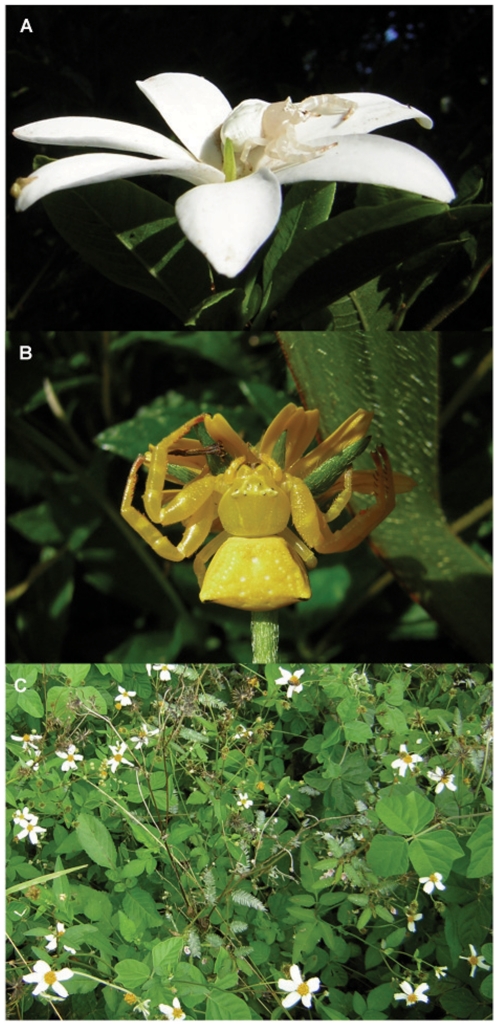
Spiders and inflorescences used in Experiment 1. (A) A white female *Thomisus spectabilis* crab spiders sitting on a white flower, (B) a yellow female *Thomisus spectabilis* crab spiders sitting on a yellow inflorescence and (C) a *Bidens alba* patch.

We run the experiments in May and June 2009, at roadside patches of daisies, *Bidens alba*, in the vicinity of Cannonvale (Queensland, Australia). We conducted the observations in six patches, distant at least one kilometre from each other. *Bidens alba* has white inflorescences with yellow centres (Fig. 1C), it was one of the dominant flowering species in our study site and it was commonly used by crab spiders as hunting platform. In our field sites *B. alba* inflorescences were mainly visited by honeybees, *Apis mellifera*. Our model predator was *Thomisus spectabilis*. We used white and yellow adult and sub-adult females (Fig. 1 A and B). The colour signal produced by these spiders is a plastic trait, spiders can change between white and yellow colour over several days [for other species of crab spiders see 10,14,16]. We collected white spiders from *B. alba* patches and yellow spiders from *Sphagneticola trilobata* patches. We kept spiders in plastic containers, feeding them with honeybees every week and watering them daily.

Each day we measured with a hand-held calliper the tibia-patella length and prosoma width of the spiders used that day and the reflectance spectra of spiders (dorsal side of abdomen) and inflorescences (upper side of inner and outer florets). Because tibia-patella length and prosoma width were highly correlated (P<0.0001, F_1_ = 1053.02, R^2^ = 0.906, N = 111), we used prosoma width as a measure of spider size in all analyses.

### Spider and inflorescences colour measurements

Spiders and inflorescence samples were analysed with an Ocean Optics USB4000 spectrometer using a fibre-optic probe connected to a black probe holder to exclude ambient light at an angle of 45° to the surfaces to measure (spiders or inflorescences). All the measurements were taken in the dark. The USB4000 spectrometer was connected to the PX-2 light source and attached to a PC running OOSpectra Suite spectrometer software. Reflectance data (300-700 nm) were generated relative to a white standard (Ocean Optics WS-1) and a black standard (black tape used as background to the measurements). For each sample, 10 spectra were averaged to reduce noise from the spectrometer with an integration period of 250 ms. We took in total three samples of each spider and inflorescence and averaged them to calculate the excitation values (E) that spiders and inflorescences would produce on the different photoreceptors (ultraviolet, blue and green) of honeybees following the methodology described below.

### Calculation of bee's photoreceptor excitation values (E values)

We evaluated how the spiders and inflorescences are perceived by *Apis mellifera* bees by calculating photoreceptor excitations and colour contrasts using the colour hexagon model [28], [29]. The relative amount of light (quantum catch) absorbed by each bee photoreceptor, *P_i_*, where *i* stands for UV, Blue or Green, was calculated by the formula:
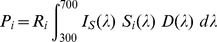
(1)where 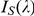 is the spectral reflectance calculated from the spiders or inflorescences; 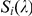 is the spectral sensitivity function of bee photoreceptor *i* and 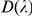 is the illuminating daylight spectrum for which norm-function D65 is employed for open habitats [provided in 30]. In equation (1) *R_i_* is the sensitivity factor, determined by the equation:
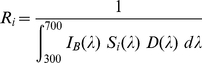
(2)where 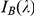 is the reflectance of the environmental background to which receptors are adapted. Note that in most conditions under which bees view flowers, the background will be green foliage, therefore, for the environmental background we used the green leaf spectrum provided by Chittka & Kevan [30].

The excitation of each bee photoreceptor, *E_UV_*, *E_Blue_*, *E_Green_*, was calculated from the relative quantum catch of the photoreceptors, *P_i_*:
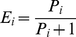
(3)


As mentioned before, the *E_UV_*, *E_Blue_* and *E_Green_* for each spider and inflorescence were calculated using the average of the excitation values calculated from the three reflectance spectra taken for each spider and inflorescence.

### Calculation of colour contrast

We used the *E_UV_*, *E_Blue_* and *E_Green_* values to calculate the colour loci of spiders and their flower background in the bee colour hexagon and estimated the chromatic contrast between each pair of spider and inflorescence by the Euclidean distance between their colour loci in the bee colour hexagon. For doing this *E_UV_*, *E_Blue_* and *E_Green_* were used to calculate coordinates in the bee colour hexagon [29], [31]:
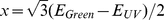
(4)

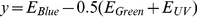
(5)


Then, the colour contrast was calculated by the Euclidian distance between the spiders and the inflorescences in the colour hexagon:
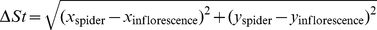
(6)where *x* and *y* are the coordinates of the hexagon calculated by equations (4) and (5).

The processing of colour information by the visual system of honeybees follows different pathways depending on the angle subtended by the visual target: when the angle is large (greater than 15°), honeybees use colour contrast to discriminate between an object and its background, but when the angle is small they use green contrast. Hence honeybees only use chromatic contrast (colour contrast, equation 6) to discriminate an object at short distances and they use the green photoreceptor (achromatic contrast) to discriminate an object from long distance [32]. In practice, this means that for our experiments colour contrast became relevant when bees were approximately less than 5–10 cm from inflorescences: According to Giurfa *et al.* [32], the relationship between the radius of an object (*r*) and the distance at which the object can be detected if it offers colour contrast with the background, *d*, is:
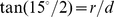
(7)


Detection distances of 5 and 10 cm therefore correspond to stimuli with radio 0.7 and 1.3 cm, respectively. An effective diameter between 1.3 and 2.6 cm is reasonable for *T. spectabilis* (average prosoma width ± SD  = 3.60±0.70).

To account for “long distance” detection, we also calculated green contrast between spiders and inflorescences as the excitation difference in the green photoreceptor between the target, spider, and the background, inflorescence. In order to describe the excitation of UV and blue photoreceptors we also calculate the specific contrast for these bee photoreceptors using the same method. Moreover, spider UV reflection has been shown to be a key factor determining the interaction between Australian crab spiders and honeybees [23]–[25], thus, we further computed the percentage of light reflected by each spider in the UV range (300–400 nm), %UV, as an absolute-value of UV reflectance, independent of assumed properties of the receiver visual system.

### Experiment 1: effect of natural spider colour on honeybee behaviour

In Experiment 1 we studied the response of honeybees to the presence of white or yellow crab spiders, *T. spectabilis*, on white daisies with yellow centres, *B. alba* (Fig. 1). In each trial we selected three nearby *B. alba* inflorescences and placed a crab spider female on one of them. We waited for the spider to adopt a hunting attitude and recorded spider behaviour (attacks and bee captures) and honeybee visits to the three inflorescences for the following 90 minutes. We defined spider behaviours as follows: attack if the spider attempted to capture the bee with its forelegs, and capture if the spider managed to capture and kill the bee. We considered that a bee visited an inflorescence when it landed on it. When spiders captured a prey, we removed it from their chelicerae with forceps and continued the observations. We completed 34 trials with white and 36 with yellow spiders, conducting observations in sunny days, between 09.00 and 15.30, when honeybee activity was high. We used each daisy and crab spider only once. If, during the observations, a spider tried to escape from the inflorescence where we had placed it, we excluded it from the experiment and started another trial with a new spider. To determine if we excluded some particularly unsuccessful hunters, we compared the excitation photoreceptor values *E_UV_*, *E_Blue_* and *E_Green_* and the colour/green contrasts calculated for yellow (N = 10) and white (N = 14) excluded spiders with the same values calculated for yellow (N = 26) and white (N = 17) spiders that successfully captured a honeybee during the experiment with independent t-tests.

We define as a “struggle” an event in which a crab spider embraces a honeybee with its forelimbs, regardless of whether the embrace ends in a successful capture or not. To determine whether the rate of honeybee visits to spider inflorescences decreased after a struggle, we performed the following analysis. We divided each trial in two temporal blocks: “early” and “late”. For trials in which we observed a struggle (N = 43), we considered as early observations from the start of the trial to the struggle, and as late observations from the struggle to the end of the trial. For trials without struggle (N = 15), early and late refer to the first and second half of the trial, respectively. For each temporal block, we calculated the rate of honeybee visits (number of visits per minute) to the spider inflorescence. We then compared these visit rates with a repeated-measures ANOVA. The dependent variable was the rate of honeybee visits to spider inflorescence, temporal block (early and late) entered in the model as the within subject factor, and struggle (“yes” if there was a struggle, “no” if there was no struggle) entered as the between subject factor.

To ascertain the factors affecting bee choice, we fitted a series of generalised linear models to the data and used Akaike Information Criterion, AIC, to select the most parsimonious model [33]. The null model assumed that honeybees visited spider harbouring inflorescences and control inflorescences with the same probability, i.e. the model assumed that the probability of visiting a spider harbouring inflorescence was p = 1/3 regardless of spider size or colour. The second simplest model assumed that spider presence affected honeybee choice, independently of any specific spider trait. The rest of models included several factors that could also affect bee choice: spider colour, size and %UV, and four indexes of colour matching between spider and inflorescence (both inner and outer florets): UV contrast (E_uv_(spider) – E_uv_(inflorescence)), blue contrast (E_b_(spider) – E_b_(inflorescence)), green contrast (E_g_(spider) – E_g_(inflorescence)) and colour contrast between inflorescence and spider. When several explanatory variables were correlated, we run alternative models with one or the other variable, but we did not include correlated explanatory variables in a single model.

For each analysis, we report in detail the most parsimonious model (the model with the lowest AIC value) and comment the differences with those models within two AIC units – when there were any such models. We used likelihood ratio tests to determine whether those factors remaining in the most parsimonious model had statistically significant effects on the probability that honeybees landed on spider-harbouring inflorescences [34]. The likelihood ratio test computes the deviance between two nested models. If the independent variables included in the more complex model, but not in the simpler model, have no explanatory value, then the deviance is expected to have a χ^2^ distribution, with as many degrees of freedom as extra parameters has the more complex model. All models assumed a binomial distribution of visits to spider-harbouring inflorescences. Thus, if *m* bees visited the patch during a trial the probability that *n* of them visited the spider-harbouring inflorescence would be given by the binomial distribution,
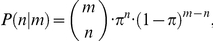
where the probability that an individual bee landed on the spider-harbouring inflorescence, *π*, is given by the fitted statistical model. We repeated the fitting procedure with different link functions (identity, logit, probit and cloglog) to select the best-fitting relationship between independent and dependent variables. Link functions had minor effects on AIC values and did not affect the variables included in the most parsimonious model. We therefore only report the results of the best-fitting link function.

We used a similar procedure to determine the factors affecting the hunting success of spiders. The dependent variable (hunting success) had again a binomial distribution. To control for possible effects of pollinator activity, on top of the explanatory variables described above we included for these analyses the number of bees visiting the patch.

### Experiment 2: effect of artificial spider colour on honeybee behaviour

**Figure 2:**
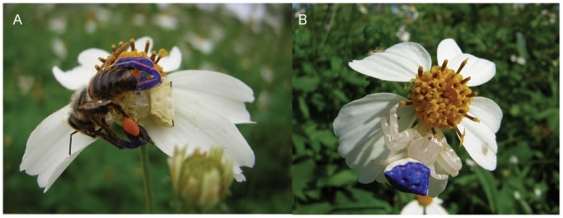
Blue painted spiders used in Experiment 2. (A) a *Thomisus spectabilis* female with the forelimbs painted on blue and (B) a *Thomisus spectabilis* female with the dorsal part of the abdomen painted on blue.

To study the reaction of honeybees towards easily detectable predators we painted some spiders with a dark-blue permanent marker. Furthermore, because it has been claimed that honeybees have a higher tendency to avoid flowers with traits resembling the shape of spider forelimbs than flowers with traits resembling the body of spiders [19], we painted in blue the forelimbs of some *T. spectabilis* females and the dorsal side of the abdomen of other females (Fig. 2). We randomly allocated white *T. spectabilis* females to one of the following treatment (37 females per treatment): “forelimbs”, “abdomen” and “control”, and painted in blue the two first pairs of legs, the dorsal side of the abdomen and the ventral side of the abdomen, respectively. The ventral side of the abdomen of crab spiders is not visible to approaching bees, but painting it served as a control for the manipulation (which could affect the behaviour of spiders) and ensured that all spiders provided the same olfactory cues. Using these three treatments rather than white and yellow spiders, we run an experiment similar to Experiment 1. In Experiment 2, however, trials lasted only 45 minutes and were discontinued when spiders struggled with a landing bee.

To determine the factors affecting honeybee choice, we used Akaike Information Criterion, AIC, to select the most parsimonious model as explained above. As with Experiment 1, the base model assumed that honeybees visited spider harbouring inflorescences and control inflorescences with the same probability, i.e. p = 1/3 for spider harbouring inflorescences. The second simplest model assumed that spider presence affected honeybee choice independently of any specific spider trait. The rest of models included some factors that could also affect bee choice: we only included treatment and spider size as possible explanatory variables in the statistical models for Experiment 2.

To determine the factors affecting the hunting success of spiders for Experiment 2, we included treatment and spider size as explanatory variables in the statistical models. The dependent variable (hunting success) had a binomial distribution. As in Experiment 1, to control for effects of pollinator activity, we added the number of bees visiting the patch as an explanatory variable in these analyses.

### Experiment 3: effect of blue spots on honeybee behaviour

To determine whether honeybees were attracted to objects presenting the blue colour that we used to paint the spiders of Experiment 2 we performed a series of observations (N = 41 trials) in which we selected four inflorescences, roughly forming a square 20–30 cm in side. We painted a blue spot on each external floret, forming roughly a circle, on two inflorescences (blue inflorescences) and the calyx of the other two (control inflorescences) to control for possible effects of ink smell. We then waited for honeybees to visit the four inflorescences ten times and noted how many of the visits had occurred on blue inflorescences. The number of times that 0, 1… 10 blue inflorescences were visited was compared to the number expected if honeybees were equally likely to visit blue and control inflorescences (binomial distribution, ten trials, p = 1-p = 0.5) using a χ^2^ test. Because the probability that blue inflorescences received 0–3 or 7–10 inflorescences was very small, and given that the χ^2^ test is unreliable if the expected number of observations in some cells of the contingency table is smaller than five, to ensure that expected values were greater than five in each cell we pooled observations corresponding to 0–3 and 7–10 blue inflorescences visited.

### Experiment 4: effect of spider movement on honeybee behaviour

We placed white *T. spectabilis* females (N = 29) on *B. alba* inflorescences, waited until they adopted a hunting attitude and recorded their behaviour with a video camera during 30 minutes. When honeybees landed on the spider inflorescence and spiders prepared to strike an attack, we gently brushed bees away to prevent captures. For all approaching honeybees, we recorded whether they landed on the spider inflorescence or rejected it. We considered that a bee rejected an inflorescence when it approached the inflorescence, hovered for a few video frames in front of it (sometimes touching it with its forelegs) and left without landing. We observed every honeybee approach frame by frame and noted the position of the spider (above or below the inflorescence) and whether it moved from the time when the honeybee entered the image until it landed or rejected the flower. We used generalised linear models to determine whether spider position and movement affected the response of the bee. The dependent variable of each model was the response of the bees (binomial error distribution: bees could either land on the inflorescence, 1, or reject it, 0), the explanatory variables were spider position (above or below the inflorescences) and spider movement (“yes” if they moved before the bee landed or “no” if the spider remained still). Spider identity was included in all models as a random factor.

Unless otherwise specified, all results are reported as average ± SE.

## Results

### Experiment 1: effect of natural spider colour on honeybee behaviour

**Figure 3:**
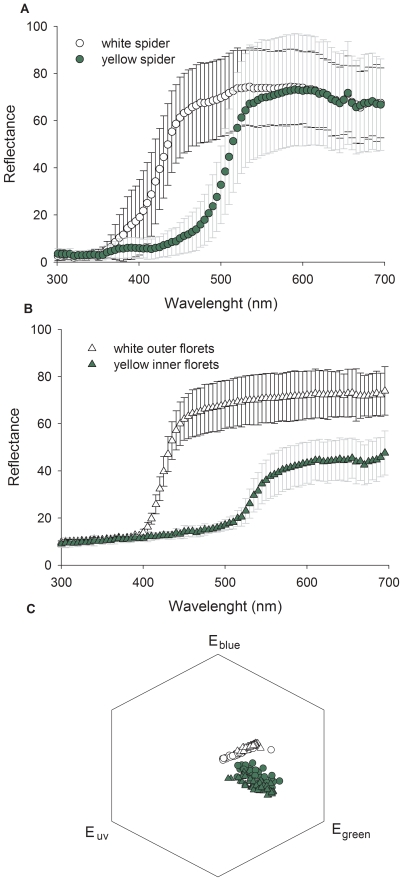
Reflectance spectra of inflorescences and spiders (Experiment 1). Reflectance spectra of (A) yellow (green circles N = 36) and white (white circles N = 34) *Thomisus spectabilis* females, (B) white outer florets (white triangles N = 34) and yellow inner florets (green triangles N = 36) of *Bidens alba* inflorescences. Error bars in panel (A) and (B) represent standard deviations. Panel C illustrates the colour loci of all spiders and inflorescences in the colour hexagon of honeybees calculated for white spiders (white circles N = 34), white outer florets (white triangles N = 34), yellow spiders (green circles N = 36) and yellow inner florets (green triangles N = 36).

Although, on the honeybee colour hexagon, there was substantial overlap between the colour loci of white spiders and outer florets of *B. alba* and between the colour loci of yellow spiders and inner florets (Fig. 3), there was variability in the loci of spiders and inflorescences and colour matching between individual spiders and the inflorescences they used as hunting platforms was generally poor. Colour contrast was 0.14±0.01 (mean ± SE, colour hexagon units) between white spiders and white outer florets and 0.17±0.01 between yellow spiders and yellow inner florets. Both values were therefore higher than the 0.05 threshold considered necessary for colour discrimination in honeybees [15]. Colour contrasts between white spiders and yellow florets (0.32±0.01) and between yellow spiders and white florets (0.27±0.01) were even easier to discriminate by the visual system of honeybees.

However, in terms of green contrast, both white (0.02±0.01) and yellow (−0.01±0.01) spiders were virtually indistinguishable from the white outer florets of *B. alba* inflorescences, but contrasted sharply (white: 0.27±0.01, yellow: 0.24±0.02) with the yellow inner florets. Taken together, these results imply that honeybees could not discriminate white or yellow spiders against the white florets of *B. alba* (where they commonly sit to hunt) when they were at large distances (more than 5–10 cm away), but they could detect the presence of the spider at closer distance, regardless of the colour of the spider or its background.

The spiders we excluded had similar colour to those we used: neither the excitation photoreceptor values *E_UV_*, *E_Blue_* and *E_Green_* nor the colour/green contrasts calculated for yellow and white excluded spiders differed from the same values calculated for yellow and white spiders that successfully captured a honeybee during the experiment (all P>0.10).

The effect of presence or absence of a struggle on the rate at which bees visited spider inflorescences was not significant (F_1,55_ = 0.55, P = 0.46), however, there was a significant effect of time in the trial (early vs. late) (F_1,55_ = 14.46, P<0.001) and the interaction between time in the trial and presence or absence of a struggle on the rate at which bees visited spider inflorescences (F_1,55_ = 11.92, P = 0.001). In trials with a struggle, the average rate of honeybee visits to spider inflorescences was 0.278 (±0.04) visits per minute before the struggle and decreased to 0.005 (±0.001) afterwards: only 11 honeybees visited a spider inflorescence after the spider struggled with another honeybee. In contrast, in trials without struggle the average rate of honeybee visits to spider inflorescences was 0.163 (±0.03) visits per minute in the first half of the observations and 0.176 (±0.03) in the second half. This result suggests that, during a struggle with a crab spider, honeybees released chemical information that elicited an avoidance response from approaching honeybees. For this reason, to ascertain the factors affecting bee choice, we only analyse honeybee visits up to and including the first struggle.

The null model assumed that honeybees visited spider inflorescences with a probability of 1/3. The model that assumed that the probability of honeybees visiting spider inflorescences was independent of spider attributes, but not necessarily equal to 1/3, provided only a slightly better fit to the data (deviance  = 2.92, df = 1, P = 0.08). According to this model, the probability of honeybees visiting spider inflorescences was 0.30. Overall, therefore, there was a modest (and not statistically significant) rejection of spider inflorescences.

The probability that honeybees landed on spider inflorescences, however, was not independent of spider attributes. According to the most parsimonious model, the probability that a bee selected the spider-harbouring inflorescence for landing was

**Figure 4:**
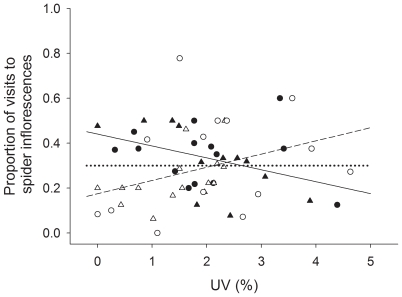
Effect of spider UV and spider size on honeybee behaviour (Experiment 1). Proportion of honeybee visits to spider inflorescences vs spider UV reflectance considering only those trials that received more than four honeybee visits to the patch. Trials with less than five visits were removed because the statistical model gives relatively little weight to trials with few honeybee visits. Black symbols represent small spiders (prosoma width <3.44 mm) and white symbols represent large spiders (prosoma width >3.44 mm). The value of 3.44 mm represents the median value of spider prosoma's width for trials that received more than four honeybee visits to the patch. Triangles represent yellow spiders and circles represent white spiders. Regression lines between proportion of honeybee visits to spider inflorescences and spider UV reflectance for small (solid line) and large (dashed line) spiders are given in the figure, together with the expected proportion of visits to spider inflorescences if honeybees treated all inflorescences alike (p = 1/3; dotted line).

*π*  = 0.74 − 0.13*spider size + 0.12*UV + 0.14*Gci + 0.049*Spider size*UV – 0.21*UV*Gci,

where *spider size* refers to spider prosoma width (in mm), *UV* to %UV reflectance of spiders and *Gci* to the green contrast generated by spiders against the inner florets of their inflorescence. The second best supported model (with a difference of less than two units in its AIC from the first model) was the model that included spider size, % UV, Gci and the double interactions between spider size and %UV, spider size and Gci, and %UV and Gci. Nevertheless, of the variables retained in both models only spider size (deviance  = 7.511, df = 1, P = 0.006) and the interaction between spider size and %UV reflectance (deviance  = 8.61, df = 1, P = 0.003) significantly affected honeybee behaviour. The probability that honeybees landed on spider-harbouring inflorescences was greatest when the spiders were large and had high UV reflectance or when spiders were small and reflected little UV, and smallest when spiders were small and had high UV or large and reflected little UV (Fig. 4). Neither Gci (deviance  = 1.26, df = 1, P = 0.26), % UV (deviance  = 0.44, df = 1, P = 0.51), nor the interaction between Gci and UV (deviance  = 2.87, df = 1, P = 0.10), nor between spider size and Gci (deviance  = 0.01, df = 1, P = 0.90) had statistically significant effects on honeybee behaviour.

Although honeybees responded similarly to the presence of white and yellow spiders, both spider colour and spider size affected the probability that a spider successfully captured a bee during the observations. The model retained to explain hunting success was:

logit(π)  = −9.39 + 5.85*spider colour + 3.25*spider size – 2.30*spider colour*spider size

**Figure 5:**
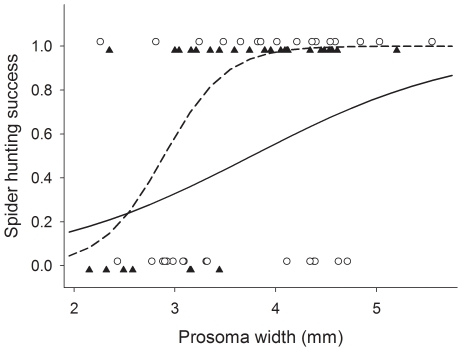
Effect of spider colour and size on spider hunting success (Experiment 1). Spider hunting success vs spider size for white spiders (white circles) and yellow spiders (black triangles). Lines represent fitted values of capture probability for white (solid line) and yellow (dashed line) spiders.

Both spider size (deviance  = 14.16, df = 1, P<0.001) and colour (deviance  = 5.59, df = 1, P = 0.018) had statistically significant effects on hunting success. Although hunting success increased with body size for both white and yellow spiders, the difference was more noticeable for yellow than for white spiders (deviance for the interaction term  = 4.60, df = 1, P = 0.032). Thus, 20 out of 20 yellow spiders with prosoma width greater than 3.44 mm successfully captured a honeybee during the observations, while only 14 out of 20 white spiders of similar size succeeded at capturing a honeybee (Fig. 5).

### Experiment 2: effect of artificial spider colour on honeybee behaviour

**Figure 6:**
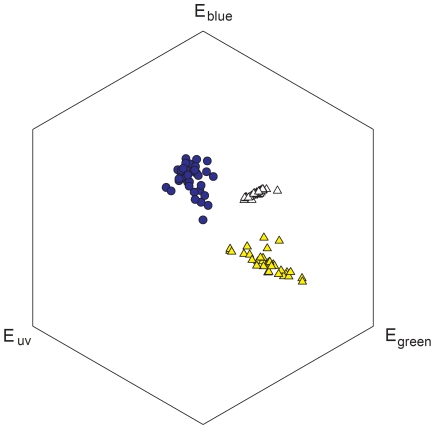
Colour loci of blue-painted spiders in the colour hexagon of honeybees (Experiment 2). Colour loci in the colour hexagon of honeybees calculated for blue-painted spiders (blue circles N = 37), white outer florets (white triangles N = 37) and yellow inner florets (yellow triangles N = 37).

As we have seen, green contrast of white and yellow spiders against the white outer florets of *B. alba* was insufficient for honeybee detection at more than 5–10 cm. The spider manipulation of Experiment 2 achieved high colour contrast (white outer florets 0.35±0.01, yellow inner florets 0.59±0.02) and green contrast (white outer florets −0.40±0.02, yellow inner florets −0.14±0.03) between the blue-painted spider traits and the inflorescences used as hunting platforms. Therefore, Experiment 2 ensured that spiders were easily detectable by honeybees at all distances (Fig. 6).

There was a significant effect of spider presence on honeybee behaviour (deviance  = 5.81, df = 1, P = 0.01). According to this result, honeybees landed on spider-harbouring inflorescences with a probability of 0.30, which was slightly lower than 1/3, therefore experiment 2 also shows that honeybees were slightly repelled by inflorescences with spiders. Only spider size (mm) remained in the most parsimonious model, according to which the probability that honeybees visiting the path landed on the spider-harbouring inflorescence, *π*, was

cloglog(*π*)  = − 0.44 − 0.17*s*pider size*

**Figure 7:**
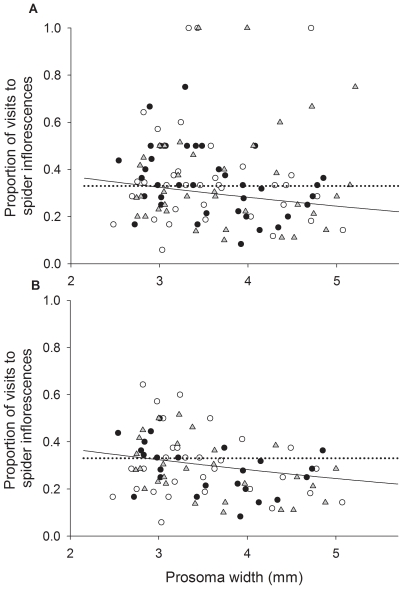
Effect of spider size on honeybee behaviour (Experiment 2). Proportion of honeybee visits to spider inflorescences vs spider size (A) considering all the trials conducted in the experiment and (B) considering only those trials that received more than six honeybee visits to the patch. Black circles represent spiders with the dorsal part of the abdomen painted on blue, grey triangles represent spiders with the forelimbs painted on blue and white circles represent control spiders. Solid lines represent fitted probability of landing on spider harbouring inflorescences. Although the relationship between probability of landing on spider inflorescences and spider size is not apparent in panel (A), the statistical model gives relatively little weight to trials with few honeybee visits removed to produce (B). Dotted lines represent the expected value if honeybees treated all inflorescences alike (p = 1/3).

**Figure 8:**
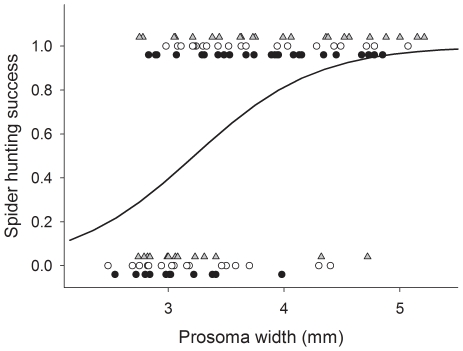
Effect of spider size on spider hunting success (Experiment 2). Spider hunting success vs spider size for spiders with the dorsal part of the abdomen (black circles), forelimbs (grey triangles) and ventral part of the abdomen (control treatment) (white circles) painted on blue. The line represents the fitted value of the probability of capturing a honeybee.

Larger spiders therefore elicited stronger avoidance responses than smaller spiders (deviance  = 5.26, df = 1, P = 0.02; Fig. 7). Treatment (deviance  = 0.48, df = 1, P = 0.78) did not appear in the most parsimonious model (ΔAIC  = 3.70). Size also affected the probability that spiders hunted a bee during the observations: large spiders posed a stronger risk for honeybees than small ones because the probability of hunting a bee greatly increased with spider size (deviance  = 28.00, df = 1, P<0.001), but treatment (deviance  = 0.81, df = 2, P = 0.66) did not enter the most parsimonious model (ΔAIC  = 3.21, Fig. 8), which was

logit(*π*)  = −6.12 + 1.90*spider size

### Experiment 3: effect of blue spots on honeybee behaviour

**Figure 9:**
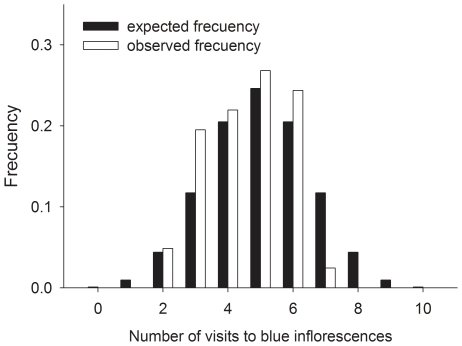
Effect of blue spots on honeybee behaviour (Experiment 3). Histograms showing the expected frequency of the number of honeybee visits to blue inflorescences when honeybees are equally like to visit control and blue inflorescences (black bars) and the observed frequency for the number of honeybee visits to blue inflorescences in Experiment 3 (white bars).

Although the results of Experiment 2 suggest that visual cues played a minor role in the predator avoidance response of honeybees, an alternative interpretation is possible. It could be argued that blue markings made spiders easier to detect, but failed to elicit an avoidance response because honeybees had a tendency to approach inflorescences with blue markings. If this were the case we should expect honeybees to be generally more attracted to objects containing blue markings compared to objects without blue markings. However the results of Experiment 3 showed that the frequency with which honeybees visited blue-painted inflorescences was not different than expected by chance (χ^2^ = 6.85, df = 4, P = 0.14, Fig. 9).

### Experiment 4: effect of spider movement on honeybee behaviour

**Figure 10:**
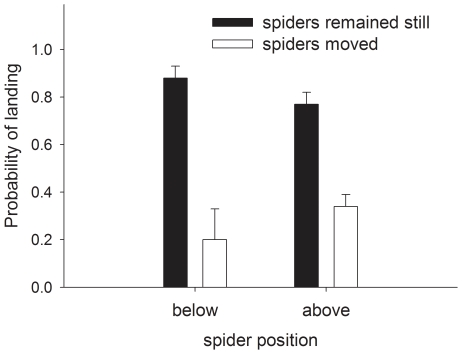
Effect of spider movement on honeybee behaviour (Experiment 4). Effect of spider movement and position on the probability (± SE) that honeybees landed on spider harbouring inflorescences.

Spider movement (deviance  = 42.64, df = 1, P<0.001) but not spider position (deviance  = 0.95, df = 1, P = 0.32) affected the probability that a bee selected the spider-harbouring inflorescence for landing. Honeybees were more likely to avoid spider-harbouring inflorescences if spiders moved during their approach rather than remaining still, and the aversive effect of spider movement was more pronounced when spiders were below the inflorescence than when they waited above it (deviance for the interaction term  = 5.25, df = 1, P = 0.02; Fig. 10).

## Discussion

The rate at which honeybees visited spider-harbouring inflorescences was not affected by the colour contrast between spiders and inflorescences, or the contrast between specific spider traits and the inflorescences. In Experiment 1 honeybees landed as often on inflorescences containing a white spider as on inflorescences containing a yellow spider. In Experiment 2 they did not discriminate between control spiders and spiders with their forelimbs or abdomen painted blue. Therefore, neither the high chromatic contrast which spiders generated against inflorescences in Experiment 1, nor the high chromatic and achromatic contrast that blue-painted spiders generated against inflorescences in Experiment 2 were sufficient to elicit a strong anti-predatory response in honeybees. These results, however, do not imply that honeybees did not respond to the presence of spiders on inflorescences: spider body size, UV reflectance and spider movement affected the rate at which honeybees visited spider inflorescences and, overall, there was a modest rejection of spider-harbouring inflorescences, which reached statistical significance in Experiment 2. According to our results, honeybees were more likely to land on spider-harbouring inflorescences when the spiders were large and had high UV reflectance or when spiders were small and reflected little UV, while other spider trait combinations elicited stronger avoidance responses. Likewise, honeybees were more likely to reject inflorescences if spiders moved as the bee approached the inflorescence than if spiders remained still.

Our results confirm a recent study which reported that colour matching between *Misumena vatia* and their flowers affected neither pollinator flower choice or spider hunting success [22]. Although the authors of this study did not measure the reflectance properties of spiders and flowers, making it difficult to assess the extent to which spiders were conspicuous to pollinators [22], we have shown that their findings remain valid when we control for the visual systems of pollinators: neither the colour contrast nor the green contrast that spiders generated against inflorescences affected honeybee response towards risky flowers. Despite the fact that our and Brechbühl *et al.* [22] study showed that spider crypsis plays a minor role in predator detection for pollinators, most of the yellow spiders that we collected in the field were collected from yellow daisies (*Sphagneticola trilobata*) and most of the white spiders were collected from white daisies (*Bidens alba*), which suggests that background colour matching may play an important role in crab spiders. One possible explanation of this finding is that crab spiders use background colour matching in response to their predators instead of their prey, but in the absence of data this possibility must be treated with caution.

Our study provides partial confirmation, under field conditions, of previous studies which suggested that Australian crab spiders exploit the plant-pollinator mutualism by creating a high UV contrast that makes flowers highly attractive for potential visitors [23]–[25]. While, in our experiments, honeybees were less deterred by large spiders with high UV reflectance than by large spiders with low UV reflectance, UV reflectance only mitigated the avoidance response, without transforming aversion into attraction. Previous work used anesthetized crab spiders and we used active spiders. Because honeybees were more likely to reject inflorescences if spiders moved as the bee approached the inflorescence than if spiders remained still, the fact that anesthetized spiders do not move may help explain the difference between our and previous results. Because previous studies had used relatively large (0.09 to 0.17 g, corresponding to 3.42–4.10 mm in prosoma width in our data set) [23]–[25] and immobilized spiders, they had only detected the positive effect of UV reflectance on bee attraction (see Fig. 4). Our study shows that the UV prey “attraction” hypothesis holds for large but not for small spiders.

Because honeybees have shared an evolutionary history with crab spiders that reflect little UV [23], it is not entirely surprising that, in the absence of UV reflection, honeybees avoid large (and dangerous) spiders but disregard the presence of small (and relatively innocuous) spiders. Spiders with naturally low levels of UV reflection in Experiment 1, and blue-painted spiders in Experiment 2, generated negative UV contrast against their inflorescences, not unlike those recorded for European crab spiders (see UV contrast in *Synaema globosum*, *Misumena vatia*, *Xysticus* sp. and *Thomisus onustus* from Herberstein *et al.* [23]). Alternatively, it is also possible that in the absence of UV reflectance honeybees avoided large but no small spiders simply because larger spiders were easier to detect. Interestingly, although honeybees could potentially behave flexibly in response to different degrees of predation threat, this behaviour only held when spiders were low UV-reflective. Higher UV-reflective spiders received, in contrast, more visits if they were large and dangerous – supporting the idea that the European honeybees have not had the opportunity to evolve a response to the deceptive UV reflective Australian crab spider.

Heiling *et al.* [18] reported that honeybees were attracted to inflorescences containing a white *T. spectabilis* female and were slightly repelled by inflorescences containing yellow *T. spectabilis* females. The apparent discrepancy between their and our results disappears if we note that Heiling *et al*. used large and immobilized spiders for their experiments, and that the yellow females they used reflected little UV light while their white females were highly UV reflective [18]. It is probably UV reflectance, and not colour (white/yellow) per se, that was responsible for the different behaviour of honeybees in their study.

If honeybees responded differently to spiders with low and high UV reflectance, it is important to point out that UV reflectance had no effect on spider hunting success. Large spiders were very successful at capturing bees and seemed to need little help of UV reflectance to capture their prey: 21 out of 39 spiders with a prosoma width larger than 3.50 mm successfully captured a honeybee within the first 15 minutes of observation. Few small spiders managed to capture bees, and given that honeybees avoided small, UV-reflecting spiders, it is difficult to imagine how UV reflectance might improve their hunting success. Thus, although UV reflectance could be beneficial in terms of hunting success when prey are scarce or when crab spiders prey on pollinators other than honeybees, our study provides little evidence that UV reflectance has evolved because UV reflecting spiders have higher intake rates.

An argument similar to the one sketched above suggests that colour matching does affect hunting success: in Experiment 1, yellow spiders were more likely to capture bees than white spiders (Fig. 5). However, we find it unlikely that the difference was due to the colour of the spiders. First of all, honeybees responded similarly to white and yellow spiders, possibly because colour contrast and green contrast were similar for both morphs. Rather, the difference may reside in the motivation of both spider groups. While running Experiment 1 we were impressed by the fact that white spiders appeared sluggish and less eager to capture bees than yellow spiders – although we realise that this is a subjective impression and difficult to quantify. Although the relationship between body mass and body size was similar for white and yellow spiders (data not shown), white and yellow spiders might be in different nutritional state: we collected white spiders from *Bidens alba* inflorescences that were commonly visited by honeybees and, therefore, honeybees may have been the main prey of white spiders. In contrast, yellow spiders were collected from *Sphagneticola trilobata* inflorescences which were hardly visited by any bee at the time of collection. Indeed, white spiders were commonly collected while feeding on honeybees, whereas yellow spiders were collected with other prey items such as crickets. It is, hence, possible that yellow spiders were more motivated to catch honeybees than white spiders because honeybees were a more valuable reward for them.

There is a final caveat concerning the generality of our results. We found that colour matching did not affect the response of honeybees to spider inflorescences and that, before the spider struggled with a honeybee, the anti-predator response of honeybees was modest at best (Figs. 4 and 7). While these results confirm those of a recent study [22], it should be pointed out that a number of previous studies report strong anti-predator responses of honeybees [e.g. 35,36]. Why do honeybees avoid crab spiders in some contexts but not in others? Honeybees seem to rely on different cues to detect predators. We have seen that size and movement affected the probability that honeybees avoided crab spiders (Fig. 10) and that honeybees appeared to avoid a chemical cue emitted by the recently attacked bee. Other studies report different mechanisms [36]–[38], and of particular relevance may be the role of learning [37], [39], as it could help explain variability between ecological contexts.

In conclusion, the degree of matching between spiders and flowers (either chromatic or achromatic contrast) and the presence of any morphological trait of the spider painted blue did not influence honeybee behaviour when choosing a flower to visit, but honeybees slightly avoided spider inflorescence, and the probability of avoidance depended on spider size, spider UV reflection and spider movement. However, although spider movement helped pollinators to show anti-predator behaviour, honeybees were more likely to avoid larger (and riskier) spiders compared to smaller (and less risky) ones only when they were not UV-reflective or reflected very little amount of UV. In contrast, UV-reflective spiders attracted more prey as spider size increased. Moreover, we found that large spiders received more honeybee visits as they increased their UV reflection and the opposite occurred for small spiders. Our study, therefore, supports the idea that Australian crab spiders deceive their preys by reflecting UV colouration only for large but not for small spiders, and highlights the importance of other cues that elicited an anti-predator response in honeybees. It is worth mentioning that, to date, studies investigating the effect of UV reflection in Australian crab spiders have found that UV reflection helps spiders to attract European honeybees to the flowers where they sit [23]–[25], but it does not help spiders to attract Australian native bees [21], [26]. Although, so far, it has been proposed that this result could be explained by the fact that in the co-evolution between crab spiders and bees, native bees have evolved an anti-predatory response towards UV reflective Australian crab spiders, an alternative plausible explanation is that the introduction of European honeybees to Australia (honeybees were introduced in Australia approximately 200 years ago [27]) has released the selection of certain spider traits, like UV reflection, that are currently present in natural populations.
